# Bone Formation with Deproteinized Bovine Bone Mineral or Biphasic Calcium Phosphate in the Presence of Autologous Platelet Lysate: Comparative Investigation in Rabbit

**DOI:** 10.1155/2014/367265

**Published:** 2014-05-27

**Authors:** Carole Chakar, Nada Naaman, Emmanuel Soffer, Nicolas Cohen, Nada El Osta, Hervé Petite, Fani Anagnostou

**Affiliations:** ^1^Department of Periodontology, School of Dentistry, Saint-Joseph University, P.O. Box 11-5076, Riad el Solh, Beirut 1107 2050, Lebanon; ^2^Laboratoire de Bioingénierie et Biomécanique Ostéo-Articulaires, UMR CNRS 7052, PRES Sorbonne Paris Cité, 75010 Paris, France; ^3^Department of Prosthetic Dentistry, Service of Odontology, Pitié-Salpêtrière Hospital, AP-HP, U.F.R. d'Odontologie, Paris 7-Diderot University, Paris, France; ^4^Department of Periodontology, Service of Odontology, Pitié-Salpêtrière Hospital, AP-HP, U.F.R. d'Odontologie, Paris 7-Diderot University, Paris, France; ^5^Department of Prosthetic Dentistry, School of Dentistry, Saint-Joseph University, P.O. Box 11-5076, Riad el Solh, Beirut 1107 2050, Lebanon

## Abstract

Bone substitutes alone or supplemented with platelet-derived concentrates are widely used to promote bone regeneration but their potency remains controversial. The aim of this study was, therefore, to compare the regenerative potential of preparations containing autologous platelet lysate (APL) and particles of either deproteinized bovine bone mineral (DBBM) or biphasic calcium phosphate (BCP), two bone substitutes with different resorption patterns. Rabbit APL was prepared by freeze-thawing a platelet suspension. Critical-size defects in rabbit femoral condyle were filled with DBBM or DBBM+APL and BCP or BCP+APL. Rabbits were sacrificed after six weeks and newly formed bone and residual implanted material were evaluated using nondemineralized histology and histomorphometry. New bone was observed around particles of all fillers tested. In the defects filled with BCP, the newly formed bone area was greater (70%; *P* < 0.001) while the residual material area was lower (60%; *P* < 0.001) than that observed in those filled with DBBM. New bone and residual material area of defects filled with either APL+DBBM or APL+BCP were similar to those observed in those filled with the material alone. In summary, osteoconductivity and resorption of BCP were greater than those of DBBM, while APL associated with either DBBM or BCP did not have an additional benefit.

## 1. Introduction

Various bone-substitute materials are used to promote bone reconstruction in oral and maxillofacial areas. They have osteoconductivity properties, as they provide a scaffold for cell adhesion and proliferation and allow bone growth on their surfaces. Deproteinized bovine bone matrix (DBBM) and biphasic calcium phosphate (BCP) are two calcium phosphate-based bone substitutes with different resorption patterns, widely used in the management of periodontal and peri-implant bone defects as well as of bone augmentation procedures [1–4]. DBBM is a bone substitute of natural origin. It is a bovine bone that undergoes a low heat (300°C) chemical extraction process by which all organic components are removed, but it maintains the natural architecture of bone and results in CaP crystals characterized as hydroxyapatite with morphological and structural properties similar to that of human bone hydroxyapatite [5].* In vivo*, it is resorbed very slowly or not at all [6]. Tested extensively* in vitro *and* in vivo*, this material has demonstrated osteoconductive properties that improve bone regeneration [7]. BCPs are synthetic bone substitutes with different ratios of hydroxyapatite HA and beta-form of tricalcium phosphate (*β*-TCP) depending on the preparation and synthesis procedure. Variations of BCP regarding HA/TCP ratio showed different patterns in biodegradation and osteoconduction [8, 9]. Some clinical studies comparing BCP (60/40 HA/TCP ratio) and DBBM regenerative potential have reported conflicting results [2–4, 10] while preclinical studies using BCP in comparison with DBBM in standardized critical-size defects are not consistent [9, 11–13].

To promote bone regeneration, the aforementioned bone substitutes are supplemented with autologous platelet concentrates such as platelet-rich fibrin (PRF), platelet-rich plasma (PRP) and platelet lysate (PL) [14, 15]. Activated platelets release several chemical compounds including various growth factors and cytokines that are involved in wound healing, angiogenesis, and bone regeneration processes. It is hypothesized, therefore, that a platelet concentrate delivered at the site of a bone defect may increase the local concentration of bioactive compounds, such as growth factors, and subsequently affect functions of bone-healing related cells. Despite the attractive rationale and the* in vitro* observed beneficial effects of platelet concentrates [16],* in vivo *preclinical [17–23] and clinical [14, 24] studies, which evaluated the efficiency of particulate bone substitutes supplemented with platelet concentrates in the treatment of intrabony defects, have provided promising but not consistent results. With the exception of the biological environment of the defect, differences in key parameters that include implanted material behavior [25], mode of preparation of the platelet-concentrates, and/or individual variability [18, 26] could explain these discrepancies. In particular, most of these studies are focused on the effects of platelets concentrates in the form of PRP, a gel obtained by treating concentrated platelets with thrombin. In this case, the observed results on bone healing might be due to formation of a fibrin clot/gel, which held material particles* in situ*, and/or to the effects of the compounds released by the activated platelets.

Alternatively, platelet concentrates are used also in the platelet lysate form (PL). Platelet lysate is prepared from PRP by freezing platelets [27].* In vitro* studies showed that PL improves the migration and/or proliferation of bone healing related cells [28–30] and is used for the clinical grade expansion of hMSC. Clinical studies suggested that it promotes oral lesions healing [31] and could be potentially useful in bone reconstructive surgery [32, 33]. However, the effect of PL in conjunction with particulate bone substitutes on bone healing has not been determined.

It is hypothesized that the mixture of bioactive molecules in the PL added to a bone substitute promotes bone healing and its effect depends on the implanted material. The objective of the present study was to compare healing around particulate DBBM and BCP and to evaluate the benefit of APL use on the potential of these bone graft substitutes to promote bone repair. To investigate these aspects, DBBM and BCP were associated with autologous PL and implanted in critical-size femoral defects, in rabbits.

## 2. Materials and Methods

### 2.1. Materials

The Materials used in the present study were as follows:

- particles (500–1000 microns in diameter) of biphasic calcium phosphate (BCP; BoneCeramic; Straumann, AGBasel, Switzerland), a composite of 60% hydroxyapatite (100% crystalline), and 40% *β*-Tricalcium phosphate, with 90% porosity and interconnected pores. This material is used as an alloplastic bone graft substitute in dental and orthopedic clinical practice;
- particles (250–1000 *μ*m in diameter) of deproteinized bovine bone DBBM (Bio-Oss; Geistlich, Switzerland), obtained from excised bone through a multistage purification process. The specimens had a porosity of 75–80% and interconnected pores. This material is a xenograft bone substitute.

### 2.2. Animals

Twelve four-month-old male New Zealand rabbits (Segav, Saint-Mars d'Egrenne, France) with a mean weight of 3.5 kg were used in the present* in vivo* study. Animal procedures were approved by the local ethics committee (Comité 4, Île-de-France, Paris, France). The animals were housed individually in metal hutches in an appropriate environment (ambient temperature of 21°C and 50% air humidity) that met the requirements of the* European Guidelines for Care and Use of Laboratory Animals* (Directive du conseil 24.11.1986, 86/609/CEE). Artificial light conditions were used in the animal facility to maintain a normal day/night biological rhythm in the animals. The animals drank water and ate commercial food concentrates (Pietrement, Sainte Colombe France)* ad libitum.*

#### 2.2.1. Preparation of Rabbit APL

Rabbit APL was prepared one week before surgery and stored according to previously established methods [18]. Briefly, G21 infusion sets (Ago Microperfusore, Artsana, Como, Italy) were used to obtain 10 mL blood from the intermediate branch of the caudal auricular vein from each rabbit. The blood samples were collected in Venoject tubes (Centravet, Plancoet, France) containing ACD-A: citric acid-dextrose-formula A (B Braun Medical S.A., Boulogne, France) (1 : 3 ACD-A (v/v)/blood) and gently centrifuged at 150 ×g and 20°C for 10 min. Aliquots (5 mL) of the platelet-rich supernatant plasma were mixed with 0.6 mL ACD-A and centrifuged at 1500 ×g and 20°C for 10 min to obtain platelet pellets. The supernatant platelet-poor plasma (PPP) was removed and stored at −80°C. The packed platelets were suspended in isotonic saline to obtain a platelet suspension of 10^9^ platelets/mL. Each APL was obtained using a series of five cycles of freezing-thawing of the respective autologous platelet suspensions followed by centrifugation at 10^4^ ×g and 4°C for 1 hour to remove platelet membranes and other cell related debris and then stored at −80°C. The biological activity of APL, with different concentrations of suspended platelets (0.01–0.3 × 10^9^ cells/mL), was also assessed* in vitro* by testing its mitogenic effect. Preconfluent foetal rat calvaria cells were incubated with rabbit APL used in the present study or not (controls) for 24 h. The cells were then removed by trypsinisation and counted using established techniques [28]. Platelet lysate enhanced, respectively, the proliferation of foetal rat calvaria cells by about 100% to 200%.

#### 2.2.2. Animal Group

The rabbits tested in the present study were randomly assigned to one of two groups, each composed of six animals. All animals underwent bilateral femoral surgery. The femoral defects in the first group of rabbits were randomly filled either with 250 mm^3^ DBBM mixed with 250 *μ*L of physiological saline (control) or with DBBM mixed with 250 *μ*L APL (with a platelet concentration of approximately 10^6^ platelets/*μ*L; lysate from 2.5 × 10^8^ platelets). The femoral defects in the second group of rabbits were filled either with 250 mm^3^ BCP mixed with 250 *μ*L of physiological saline (control) or with BCP mixed with 250 *μ*L APL. Defect fillers were prepared and used under sterile conditions at time of animal surgery. All animals were sacrificed 6 weeks after implantation.

### 2.3. Surgical Procedures

#### 2.3.1. Anesthesia

Prior to surgery, the rabbits were anesthetized using an intramuscular injection of 0.5 mg/kg Diazepam (Valium; Roche; Basel, Switzerland), 0.25 mg/kg metedomidine hydrochloride (Domitor, Virbac; France), and 100 mg/kg Ketamine hydrochloride (Ketalar 500, Pfizer; France). The anesthetized animals were prepared for aseptic surgery, shaved, and disinfected; both lower limb sites were draped using standard sterile procedures.

#### 2.3.2. Surgical Procedure

The surgical technique used was adapted from the one described by Soffer et al. [18]. Briefly, a longitudinal skin incision was made to expose the distal lateral aspect of each femoral condyle. A circular cortical window was formed using a 6 mm wide internal diameter trephine. A cylindrical critical-size defect of 10 mm deep and 5.5 mm wide was then created in the lateral condyle in a stepwise fashion using color-coded drills, (Diffusion International, Paris, France). These cavities were thoroughly rinsed with isotonic saline to remove bone debris and then, using a specially designed injector and applying a light pressure, they were filled with DBBM or BCP mixed with either physiological saline or APL. The aforementioned fillers were prepared and used under sterile conditions at the time of animal surgery. Each soft tissue wound was closed in two successive layers soft tissue and skin, respectively, using resorbable Vicryl 5/0 sutures, and the surgical site was disinfected.

#### 2.3.3. Postsurgical Animal Treatment

All animals were given intramuscular injections of 0.2 mg/kg metoxicam (Metakam Boehringer Ingelheim Vetmedica; GmbH Germany) to relieve pain during the postoperative 24 h period. Prophylactic antibacterial treatment, consisting of 25 mg/kg animal weight sulfadimethoxine trimethoprim (Copylap Biové; France), was also administrated for 5 days after surgery by qualified animal-care staff. After surgery, all operated animals were monitored until they were able to walk on their own. The rabbits were sacrificed using an overdose of pentobarbital 6 weeks after implantation. At that time, the femoral condyles were removed and cleared of the surrounding soft tissue. All excised bone tissue specimens were prepared for histological analysis as described in the histology section.

### 2.4. Histology

All excised specimens were examined histologically using an established procedure for nondemineralized bone [23]. Each excised bone specimen was cleared of soft tissue, fixed in 10% phosphate-buffered formalin, rinsed in water, dehydrated in ethanol, cleared in xylene, and embedded in plastic resin (Technovit 7200 VCL, Kulzer & Co. GmbH, Wehreim, Germany). Radiographs were taken to ensure appropriate defect orientation for subsequent histologic sectioning. The femoral condyles were sectioned perpendicular to the long axis of each defect. Each section was then ground down to a thickness of 100 *μ*m using the Exact Grinding System (Exact Aparatebau GmbH Norderstedt, Germany) and polished down to 80 *μ*m. All tissue specimens were then stained using Giemsa-Paragon for histomorphometric analysis.

### 2.5. Histomorphometry

**Figure 1:**
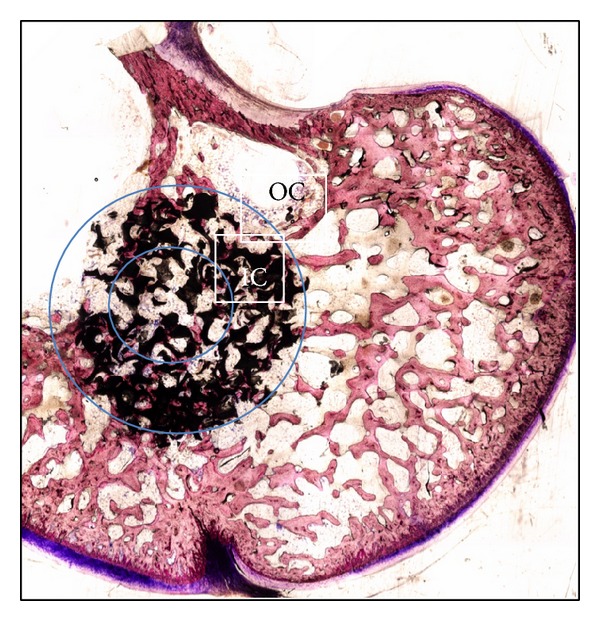
Schematic representation of a condyle section. The newly formed bone and the residual material distribution were evaluated separately in the inner (IC) and outer zones (OC) at 6 weeks after implantation.

Three randomly selected sections per condyle and per animal were analyzed. Each section was examined under a light microscope (Olympus BX 60, Olympus Corporation, Tokyo, Japan) connected to a digital camera (Olympus, E330). New bone formation was quantified using Image Tool 3.0 software (UTHSCA, SanAntonio, TX, USA). The spatial calibration of the histological sections was done by drawing manually the surface of the newly formed bone and the residual bone substitutes. The total surface was obtained by adding all the delimited areas measurements (in mm^2^). The distribution of newly formed bone and residual implanted material was also determined by classifying the defect area into an inner zone (IC) of 2.5 mm and an outer zone (OC), including the remaining of the 5.5 mm defect (Figure 1).

### 2.6. Statistical Analysis

Two-way analysis of variance between groups was conducted to determine the effect of bone substitutes with APL on new bone formation and on the resorption of these materials. Variables were tested for normal distribution and equality of variance using the Kolmogorov Smirnov and Levene tests, respectively. Comparison of the data between the different groups tested was performed using the Student's *t*-test. All statistical analyses were performed using commercially available software programs (SPSS for Windows version 16.0). Significance was defined as a *P* value of less than 0.05.

## 3. Results

### 3.1. Histological Observation

All rabbits recovered within 2-3 hours after surgery. In all cases, tissue healing was uneventful. Rabbits were systematically checked in order to detect any problems during the experimental period and they were weighed every two weeks. No postsurgical complications, infections, animal behavior, or general health changes were observed after surgery and for the duration of the study. Rabbits presented a normal weight gain of 103,8 g.

**Figure 2:**
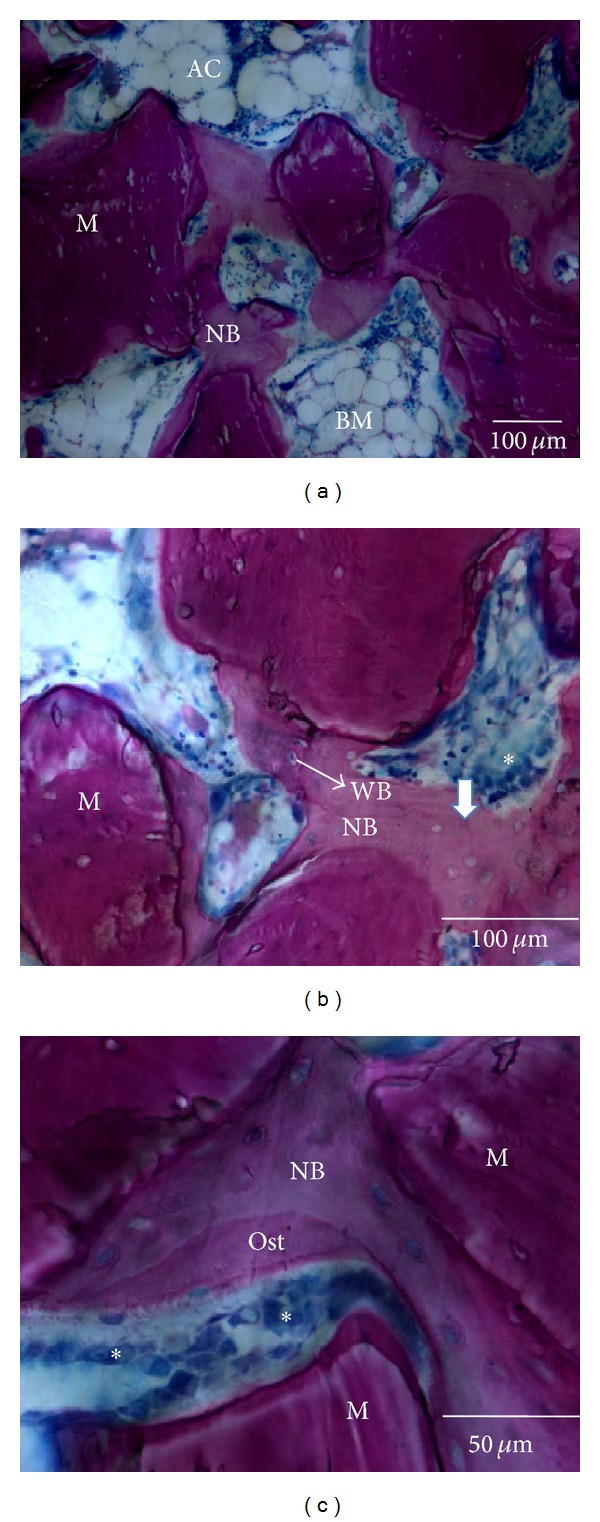
Representative light micrographs illustrating histological details of a defect grafted with DBBM at 6 weeks after implantation. DBBM (M) surrounded and bridged by newly formed bone (NB). The increased number of adipocytes (AC) in the wide marrow spaces (BM) indicates bone marrow maturation ((a); magnification ×10). Woven bone (WB) and mature paralleled-fiber bone (arrows) were observed ((b); magnification ×20). Osteoblast-lining cells (asterisks) on the osteoid surface (OST) indicate ongoing bone formation ((b); magnification ×20 and ((c); magnification ×40). Stain: Giemsa-Paragon.

**Figure 3:**
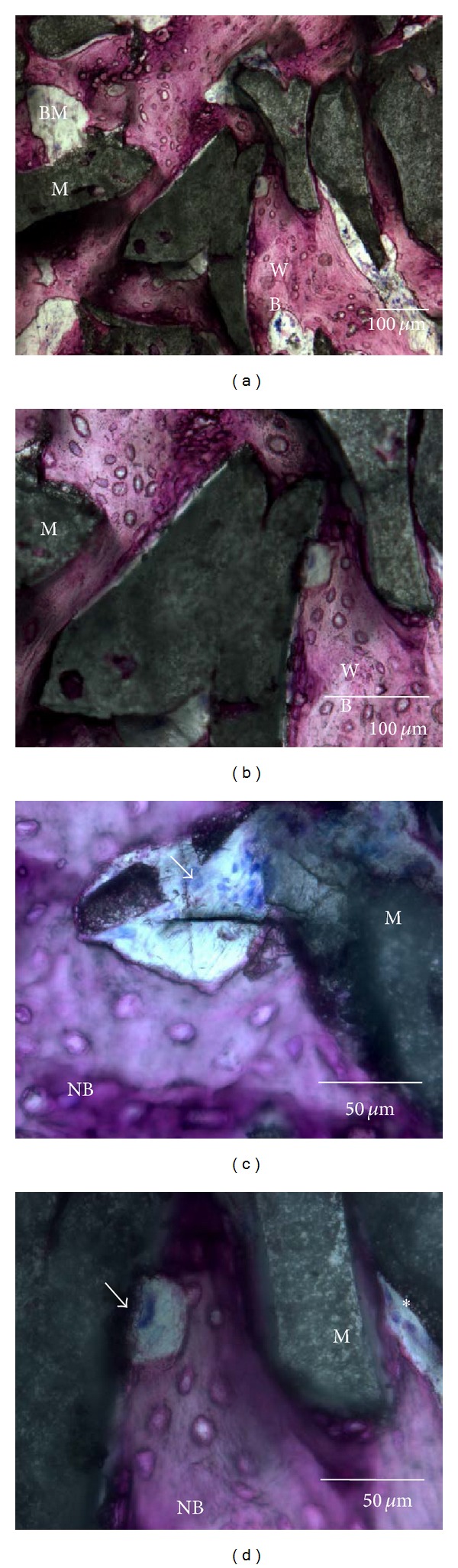
Representative light micrographs illustrating histological details of a defect grafted with BCP at 6 weeks after implantation. BCP surrounded and bridged by newly formed bone ((a); magnification ×10 and (b); magnification ×20)). Mostly newly formed bone had woven-bone (WB) characteristics (b); giant multinuclear cells (arrows) were observed on the BCP particles ((c) and Frame (d); magnification ×40). Stain: Giemsa-Paragon.

After 6 weeks of implantation, histological analysis of specimens excised from all animals tested showed no inflammatory reactions. New bone formation was evident in close contact to both the BCP and DBBM particles that have been used to fill the femoral defects in the present study, even in the central part of the defect (Figures 2(a) and 3(a)).

In the defects filled with DBBM, newly formed trabecular bone with wide marrow spaces and residual particles were present. The DBBM particles were distributed over the entire grafted area of the femoral defects and were surrounded and bridged by newly formed bone (Figure 2(b)). Neither gaps nor connective fibrous tissues were present at the bone biomaterial interface. A thin layer of osteoid and osteoblast lining-cells was observed in some areas around DBBM particles (Figures 2(b) and 2(c)). Multinucleated giant cells or Howship lacunae, pits, or resorptive trails were not seen on the DBBM surface regardless of the presence or absence of APL. The DBBM thus exhibited osteoconductive properties but not ongoing material resorption.

In the defects filled with BCP, the material particles were distributed over the entire defect area and they were surrounded by newly formed bone (Figure 3(a)) both in the presence and absence of APL addition. Some particles were covered by a layer of newly formed bone (Figure 3(b)). Sites where the material had been resorbed had new bone and marrow spaces. Some lining osteoblasts, as well as multinuclear phagocytic cells, were observed at the surface of the BCP particles (Figures 3(c) and 3(d)). In some areas, particles were embedded in bone; in such cases, osteocytic lacunae were observed. Examination at high magnification (×40) revealed that the most newly formed bone had woven bone characteristics. New bone formation was more prominent in the outer circle (OC), bridging with the edges of the defects, than in the inner circle (IC).

### 3.2. Histomorphometric Results

**Figure 4:**
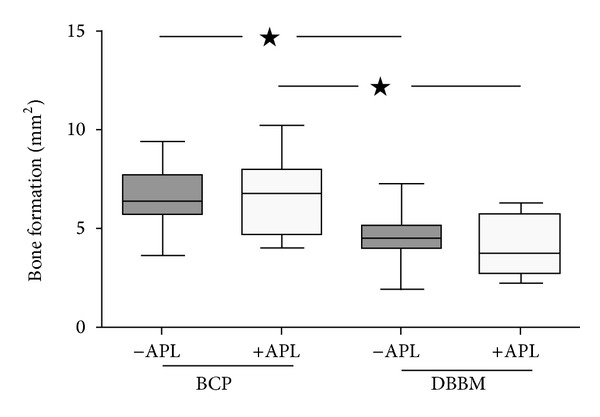
Box plot showing bone formation area in the defect at 6 weeks after implantation. In the defects filled with BCP, the newly formed bone area was significantly higher than that observed in the defects filled with DBBM (*P < 0.001). No significant differences were observed between defects filled with BCP and BCP+APL, as well as between defects filled with DBBM and DBBM+APL.

**Figure 5:**
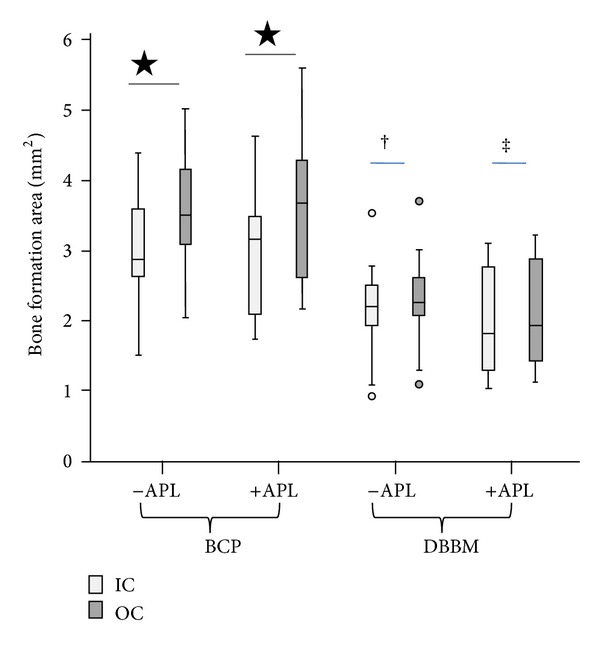
Box plot showing bone formation distribution throughout the defect at 6 weeks after implantation. For the two filling materials, the bone surface areas in the outer zone (OC) were significantly higher than those observed in the inner zone (IC) of the defects (for BCP *P < 0.0001 in the presence and absence of APL; for DBBM ^†^ *P* < 0.001 and ^‡^ *P* < 0.037 in the absence and presence of APL).

At 6 weeks after implantation, the bone areas in defects filled with BCP were similar to those observed in defects filled with BCP+APL (6.69 ± 1.53 mm^2^ and 6.57 ± 1.90 mm², respectively; Figure 4). The amount of bone formed in defects filled with either DBBM or with DBBM+APL was also similar (4.49 ± 1.19 mm^2^ and 4.28 ± 1.42 mm^2^, resp.). The addition of APL did not promote new bone formation above the extent obtained with the two bone substitutes tested alone. In contrast, the bone areas in defects filled with BCP were significantly (*P* < 0.0001) higher than those observed when DBBM alone was used as the filler. Specifically, the mean difference in the bone amount in defects filled with either BCP or DBBM was 2.29 mm^2^ (confidence interval (CI) 1.23–3.36) in the absence of APL and 2.19 mm^2^ (confidence interval (CI) 1.13–3.25) in the presence of APL. For the two substitute materials as well as for all of the experimental conditions tested in the present study, the bone surface areas in the outer zone (OC) were significantly higher than those observed in the inner zone (IC) of the defects (for BCP: *P* < 0.0001 in the presence and absence of APL; for DBBM: *P* < 0.037 and *P* < 0.001 in the presence and absence of APL; Figure 5). Distribution of newly formed bone throughout the respective original defect was not affected by the presence or absence of APL. The bone surface area at the inner as well as at the outer zones of the defects was significantly (*P* < 0.001) higher when the defects were filled with BCP than when they were filled with DBBM (Figure 5).

**Figure 6:**
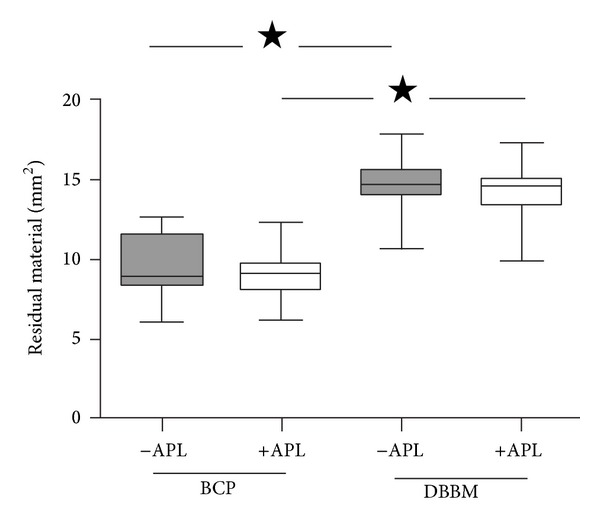
Box plot showing residual material area in the defect at 6 weeks after implantation. In the defects filled with DBBM, the amount of residual material was significantly (*P < 0.0001) higher than that observed in the defects filled with BCP.

**Figure 7:**
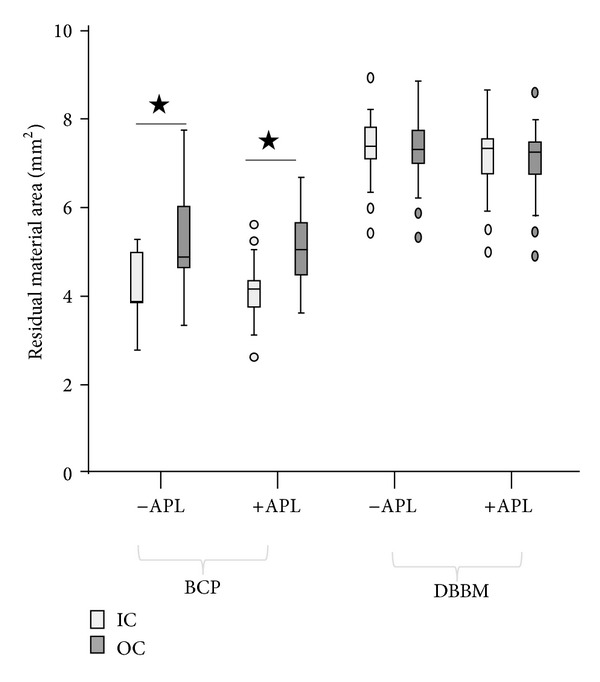
Box plot showing residual material distribution throughout the defect. In the defects filled with BCP, the amount of residual material was significantly (*P < 0.0001) higher in the outer zone (OC) than in the inner zone (IC) of the defect. In contrast, the amount of residual material present in the defects filled with DBBM was similar in the inner and the outer zones.

At 6 weeks after implantation, the surface areas of the residual implanted material in defects filled with BCP was similar to those in defects filled with BCP+APL formulations (9.43 ± 1.89 mm^2^ and 9.25 ± 1.61 mm^2^, respectively; Figure 6). In addition, the surface areas of the residual material in defects filled with DBBM was similar to those filled with DBBM+APL formulations (14.61 ± 1.64 mm^2^ and 14.36 ± 1.93 mm^2^, resp.). The amount of implanted material in defects filled with BCP was significantly (*P* < 0.0001) lower (specifically, close to 65%) compared to that observed with DBBM regardless of the presence of APL. Analysis of the distribution of the nonresorbed implant material throughout the defect provided evidence that, in the defects filled with BCP, the amount of residual material was significantly (*P* < 0.0001) higher in the outer zone than in the inner zone of the defect (mean difference 1.04 mm^2^; confidence interval (CI): 0.70–0.84; Figure 7). Similar results were observed in the defects filled with BCP+APL formulations (mean difference 0.95 mm^2^; *P* < 0.0001; confidence interval (CI): 1.11–0.79). In contrast, the amount of residual material present in the defects filled with DBBM in either the presence or the absence of APL was similar in the inner and the outer zones (mean difference 0.06 mm^2^; *P* < 0.43; confidence interval (CI): 0.09–0.21 and 0.07 mm^2^; *P* < 0.368; confidence interval (CI): 0.08–0.22 with and without APL, resp.).

## 4. Discussion

This study compared the osteoconductive and resorption behavior of BCP and DBBM alone or associated with APL, in critical-size rabbit femoral defects. Rabbit is a validated model, currently used in bone reconstruction research. Its size and development level allow performing easy surgical procedures [34]. In particular, the femoral site is an interesting site for the evaluation of the biofunctionality of biomaterials because of the equilibrium of the osteogenesis and the biodegradation activities [35]. In order to achieve standardization in the evaluation of bone substitutes, different authors have developed a critical-size defect model. A critical-size defect is defined as the smallest intraosseous wound that does not heal by bone formation during the lifetime of the animal [36, 37]. According to international standards (ISO 10993-6), bone substitute materials must be evaluated in bone defects of at least 2 mm diameter and 6 mm in length for rabbit femurs. DBBM and BCP are the most widely used grafting materials in oral, maxillofacial, and implant surgery [1, 7] and they are frequently used associated with platelet concentrates [14, 24]. However, there are few experimental studies in the literature focusing on the evaluation of their regenerative potential using standardized intrabony defects within the same study [11] and, to our knowledge, none has used critical-size femoral defects to compare the regenerative properties of these bone substitutes.

Results obtained in this study showed the presence of newly formed bone in close contact with the particles of DBBM and BCP as reported in previous preclinical [9, 12, 13] and clinical studies [2–4, 10]. Regarding the distribution of newly formed bone, between the inner and outer zones, new bone formation was more prominent in the OC than in the IC, which shows a slight tendency of augmented bone formation in the outer zone of the defect. Other histomorphometric studies have suggested that the majority of the bone formation starts from the borders of the defect, as a centripetal bone colonization [38]. Interestingly, under the experimental conditions of the present study, significantly (*P* < 0.001) more bone formation was observed in the BCP group (close to 70%) after 6 weeks implantation time in the presence or absence of additional APL (Figure 4). A similar tendency was described in standardized intrabony defects when BCP was compared to pure hydroxyapatite in the mandible of minipigs at 4 and 8 weeks after implantation [9]. In contrast to the results of the present study, BCP and DBBM rendered similar amount of newly formed bone in noncritical size intrabony defects in the mandible of minipigs at 4 and 8 weeks after implantation [11], in the rabbit calvaria [12] as well as around implant placed in circumferential defects in dogs mandibles at 8 weeks after implantation [13]. The reasons for these differences are not clear; however, the anatomical and dimensional characteristics of the defects sites (femur in the present study versus calvaria) [12] or mandible [11] and the duration of the study (6 weeks in the present study versus 4, 8, and 16 weeks in others) [11, 12]) may be contributory factors. The efficiency of BCP could be related to its partial dissolution and the enhanced bioactivity of soluble TCP [39]. Further studies with different healing intervals are necessary to clarify the differences between DBBM and BCP osteoconduction efficiency as well as the characteristics of the newly formed bone in their presence.

The most striking difference between the two bone substitutes tested in the present study was their resorption behavior. For the same volume of implanted material, the amount of BCP residual particles in the defect was significantly (*P* < 0.001) lower (specifically, close to 65%) than that observed in defects filled with DBBM at 6 weeks after implantation. In contrast, a limited resorption was observed in mandible bone defects in minipigs [11] and no BCP resorption in calvaria bone defects in rabbits at 4 and even at 16 weeks after implantation [12]. This disparity may be explained by the differences in the anatomical site of the defect; calvaria is a site where blood supply and bone marrow are poor. However, the results of this work are in accordance with previous studies that reported also approximately 50% resorption of BCP (60/40 HA/TCP ratio) in osseous defects in rabbits within one year after implantation [40]. The resorption of implanted BCP particles provided space and may explain the higher new bone formation observed with this material. Comparison of the distribution of the nonresorbed material throughout the defect showed that, in the defects filled with BCP, the amount of the residual material was significantly (*P* < 0.001) higher in the outer zone than in the inner zone of the defect. This finding may be correlated to the greater bone formation observed around BCP particles in continuity to the surrounding bone. Regarding the DBBM resorption, residual material in defects filled with DBBM was significantly (*P* < 0.001) higher than that observed in defects filled with BCP, indicating that DBBM resorption is very slow [7]. Moreover, under the experimental conditions of the present study, multinucleated cells were not observed on the DBBM particles in line with previous findings [6].

Since the use of high local concentrations of platelet-derived soluble compounds with bone substitute material remains an attractive strategy for improving bone repair given its autologous origin, minimally invasive nature, and cost-effective ratio, but that it has not provided consistent results, the present study was motivated to explore bone healing in defects filled with either DBBM or BCP in the presence of APL. The results obtained at 6 weeks after implantation in the rabbit femoral defects showed that osteoconduction of BCP particles was more pronounced than in the case of DBBM, and the addition of PL did not affect those outcomes. A direct comparison between the results of the present study and published reports on platelet-derived bioactive components is difficult because of the differences in the platelet preparation (PRP, PRF, and APL) and/or in the study design [26]. Nevertheless, the findings of the present study corroborate those of other animal studies, which also showed that PRP in conjunction with DBBM in critical-size rabbit calvaria [21] and frontal defect of goat [17, 20] does not improve DBBM osteoconductivity. As regards BCP, PRP in combination with BCP (ratio 60/40) was tested only in rat calvaria defects [19, 23] and no positive effect of PRP was established. However, recently it has been described that PL coating of BCP increases* in vitro* chemoattraction and adhesion of MSCs and improves* in vivo* neovascularization and new bone formation [33].

Moreover, it has been suggested that platelet concentration plays a role in the effect of platelet concentrates in bone healing. In the present study, APL with a platelet concentration of 10^6^ platelets/*μ*L (3.5 to 5X fold of baseline) was used. With a similar concentration, previous studies have shown biological effects using rabbit PL [28] or rabbit PRP [41] APL with a concentration of 10^6^ platelets resulting in higher resorption of calcium carbonate ceramic in rabbit femoral defects [18]. PRP with platelet concentration ranging from 9.5 × 10^5^ to 1.7 × 10^6^ platelets/*μ*L showed improved peri-implant bone regeneration in rabbits [41]. Below this range the effect is suboptimal; beyond this range, there may be a parodoxical inhibitory effect. The time period during which platelet growth factors remain active [42] and the bioavailability of platelet components in relation to DBBM and/or BCP dissolution in osseous defect could explain the absence of platelet concentrates effect on tissue repair. These hypotheses were not explored in this work, and further investigations may be needed to optimize the use of platelet concentrates. It is not clear how far the results of this rabbit study can be extrapolated to different species. Nevertheless, previous studies reported biological effects using autologous rabbit as well as miniature pig, dog, and human PL.

Moreover, the quantitative histomorphometric measurements performed in this study demonstrated that the addition of APL did not affect the resorption of either DBBM or BCP. However, previous studies reported that platelet concentrates increase the resorption of carbonate phosphate [18] and carbonated apatite calcium phosphate particles [43] probably by triggering anti-inflammatory reaction and/or biological responses in the early events of healing [44]. In contrast to DBBM, that did not or slowly resorbed, and BCP that resorbed mildly, calcium carbonate is a material with high and fast resorption. The discrepancy observed in the resorption pattern of these materials, related to the material surface properties and the PH of the biological environment, could influence the adsorption and the release of PL adsorbed proteins and growth factors.

In summary, this study demonstrated the osteoconductive properties of these two biomaterials in critical-size osseous defects and showed in a quantitative way that compared to DBBM, BCP was resorbed and promoted a greater bone formation while APL addition had no impact on those outcomes. Results obtained in the present investigation are, therefore, relevant to the contribution of BCP and DBBM particles, used either alone or in conjunction with autologous bioactive compounds released from platelet concentrates to promote intrabony defects repair.
